# The Study on Fatigue Crack Growth Rate of 4130X Material under Different Hydrogen Corrosion Conditions

**DOI:** 10.3390/ma17010257

**Published:** 2024-01-03

**Authors:** Shaolei Jiang, Jing Wang, Bo Zhao, Enfeng Zhang

**Affiliations:** 1 Faculty of Materials and Manufacturing, Beijing University of Technology, Beijing 100124, China; shaolei9805@163.com (S.J.); z76018@emails.bjut.edu.cn (E.Z.); 2 China Special Equipment Inspection and Research Institute (CSEI), Beijing 100026, China; 3 Key Laboratory of Special Equipment Safety and Energy-Saving for State Market Regulation, Beijing 100048, China

**Keywords:** 4130X steel, hydrogen sulfide corrosion, corrosion fatigue crack growth rate, molecular dynamics

## Abstract

In this paper, the fatigue crack growth rates of typical pressure vessel material 4130X under different corrosion conditions are investigated, and the effects of corrosion modes and loading frequency on the fatigue crack growth rate of 4130X are discussed. The results show that under the same loading conditions, the pre-corroded crack propagation rate is increased by 1.26 times compared with the uncorroded specimens. The plastic deformation mechanism of the crack tip in air is dominated by phase transformation but the hydrogen introduced by pre-corrosion causes a small number of dislocations at the crack tip. The crack growth rate obtained by corrosion fatigue is four times that of the uncorroded specimen, and the fracture surface shows a strong corrosion effect. The molecular dynamics simulation shows that the hydrogen atoms accumulated at the crack tip make the plastic deformation mechanism dominated by dislocation in the crack propagation process, and the coupling interaction between low frequency and the corrosion environment aggravates the hydrogen embrittlement of the crack tip. In the air condition, the loading frequency has no obvious effect on the crack growth rate: when the frequency decreases from 100 Hz to 0.01 Hz and other conditions remain unchanged, the fatigue crack growth rate increases by 1.5 times. The parameter *n* in the Paris expression is mainly influenced by frequency. The molecular dynamics simulation shows that low frequency promotes crack tip propagation.

## 1. Introduction

Hydrogen is a clean energy that has recently gained traction. Hydrogen-blended natural gas is one of the important application scenarios of hydrogen. In recent years, high- pressure vessels have been used for storing and transporting hydrogen-blended natural gas. During the working process of the high-pressure vessel, with natural gas filling and releasing continuously, the vessel is in a typical fatigue loading condition. High-pressure hydrogen gas, and impurities such as hydrogen sulfide, water, and carbon dioxide in natural gas may also cause hydrogen embrittlement issues. The vessel is challenged by the working condition of low-frequency corrosion fatigue, which leads to high risk.

Corrosion fatigue is a common problem in engineering. The research on the crack growth rate under the corrosion fatigue condition is increasing. The fatigue crack growth rate of metals is mainly affected by the corrosion environment and loading conditions [1,2]. It has been shown that the existence of hydrogen can lead to hydrogen embrittlement [3] and reduce the fracture toughness of materials [4]. Hence, the fatigue crack growth rate of metal in hydrogen is much higher than that in the air [5,6]. The hydrogen sulfide contained in natural gas may act as a “toxic agent”, which makes the hydrogen atoms precipitated by the cathode reaction difficult to combine into hydrogen molecules to escape, so hydrogen is enriched on the surface of the steel and forms hydrogen diffusion [7,8,9], which causes great damage to the steel. Furthermore, the corrosion fatigue crack propagation rate is also affected by loading factors. As the frequency of fatigue loading decreases, the rate of metal crack propagation increases [10].

Molecular dynamics simulation can be used to reveal the mechanism of crack initiation and propagation. The simulation result shows slip band formation, dislocation emission, and crack nucleation at the grain boundaries, as well as vacancies and other key information. In recent years, the study of the fatigue crack propagation mechanism by molecular dynamics simulation has gradually increased [11,12,13]. Dogan used LAMMPS to study the tensile response of 4340 steel in a hydrogen environment. He found that the presence of hydrogen promoted the martensitic transformation of 4340 steel, resulting in local plasticity [14]. Xiao et al. investigated the effect of hydrogen atoms on the size of the plastic deformation zone. A per-charged hydrogen molecular dynamics model was used to quantify the compression effect of hydrogen atoms on the plastic zone during cyclic loading, and a modified fatigue crack growth rate (FCGR) model, combined with the compression factor, was then used to predict the hydrogen-assisted fatigue crack growth rate [15].

4130X steel, a typical material of a hydrogen-blended natural gas pressure vessel, is studied in this paper. Generally, 4130X is composed of iron atoms (α-Fe) arranged in a body-centered cubic lattice. Firstly, FCGR experiments were carried out under different hydrogen corrosion states and different frequencies, the change law of FCGR was studied, and the influence of different factors on crack growth was determined by observing the fatigue fracture morphology using SEM. Secondly, in order to study the effect of hydrogen atom distribution on the crack initiation and fatigue crack propagation behavior of α-Fe, the molecular dynamics method was applied to simulate the fatigue crack growth in the local region of the crack tip under different hydrogen-containing conditions. It provided a basis for the long-term service of 4130X steel in a hydrogen-blended natural gas environment.

## 2. Materials, Experimental Procedures, and Simulations

### 2.1. Preparation of Materials

4130X steel for hydrogen-blended natural gas cylinders was researched in this paper. The main chemical composition of 4130X is shown in Table 1, and the basic mechanical properties are shown in Table 2.

**Figure 1:**
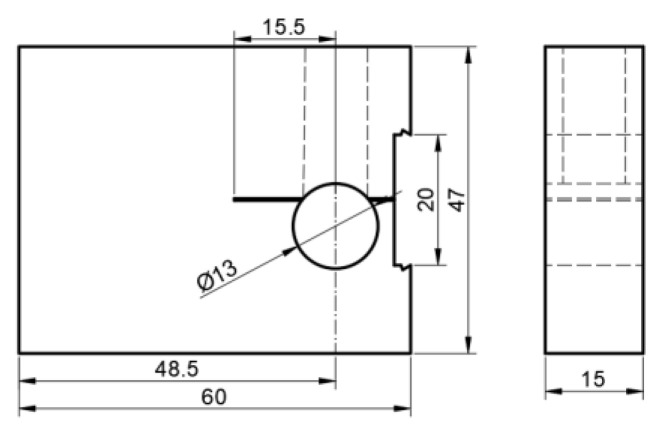
Improved WOL specimen (unit: mm).

According to GB/T 15970.6-2007 “Corrosion of metals and alloys—Stress corrosion testing—part 6: Preparation and use of pre-cracked specimens for tests under constant load or constant displacement” [16], an improved WOL specimen was selected for the experimental study. Dimensions of the specimen are shown in Figure 1.

### 2.2. Experimental Solutions

In order to study the effects of hydrogen corrosion conditions on the fatigue crack growth of 4130X material, three different hydrogen corrosion conditions were set up. Firstly, to obtain the basic properties of the material, high-frequency and low-frequency fatigue crack growth experiments were carried out in the air. Secondly, to obtain the fatigue crack propagation behavior of material, the condition of the uniformly distribution of hydrogen atoms was simulated by pre-corrosion. To study the migration of hydrogen through stress-induced action, the hydrogen content in the specimen was controlled. Finally, to obtain the law of change of the crack growth rate under the coupling of external load and the corrosive medium, the material’s actual working condition was simulated by conducting the fatigue crack growth experiment in the corrosive environment.

The class A corrosion solution recommended by NACE was used as a H_2_S corrosion environment. In the Class A corrosion solution, H_2_S gas was introduced into the aqueous solution containing 5% NaCl and 0.5% glacial acetic acid so that the concentration of H_2_S in the solution reached 400 ppm. In the pre-corrosion experiment, the improved WOL specimen was soaked in the above solution for 48 h. In this process, hydrogen entered the metal through adsorption, dissolution, and diffusion. After reaching the specified pre-corrosion time, the specimen was taken out from the corrosion environment, wiped, and immediately tested in the air, greatly avoiding the phenomenon of hydrogen escape. The corrosion fatigue experiment is the fatigue crack growth test in the corrosion loading conditions of 400 ppm.

Considering the actual working conditions of the high-pressure cylinder, the next filling took place after the high-pressure gas in the cylinder was completely emptied, and the interval between the two filling times was relatively long. The cylinder was in an extremely low-frequency fatigue condition. The stress ratio of fatigue experiments was set to 0.1 in this paper. The frequency of the pre-corroded fatigue crack growth experiment was 100 Hz in order to prevent the hydrogen escaping due to too long an experimental time, and the frequency of the corrosion fatigue crack growth experiment was 0.01 Hz, which simulates the actual working condition. At the same time, a set of uncorroded crack growth rates were tested at each frequency to form a good contrast.

### 2.3. Experimental Procedure

The test was carried out according to GB/T 6398-2017 “Metallic materials—Fatigue testing—Fatigue crack growth method” [17]. The crack opening displacement *V* of each cycle was measured using a clip gage, and the compliance equation (Equation (1)) was used to obtain the crack length *a_i_* at each moment.

(1) $$\frac{\Delta V_{i}}{\Delta P_{i}}=\frac{C_{6}\left(a_{i}/W\right)}{EB}$$

*V* crack opening displacement, Δ*V* = *V_max_* − *V_min_*, mm;
*P* load, Δ*P* = *P_max_* − *P_min_*, kN;
*B* specimen thickness, mm;
*E* elastic modulus, GPa;
*a_i_* crack length per cycle, mm;
*W* specimen width, mm;
*C*_6_ dimensionless flexibility, function of *a_i_*/*W*, see Equation (2).

(2) $$C_{6}\left(\frac{a_{i}}{W}\right)=e^{\left[4.495-16.130\left(\frac{a_{i}}{W}\right)+63.838{\left(\frac{a_{i}}{W}\right)}^{2}-89.125{\left(\frac{a_{i}}{W}\right)}^{3}+46.815{\left(\frac{a_{i}}{W}\right)}^{4}\right]}$$

After the crack length *a* of each cycle was calculated, the *a*-*N* curve was drawn and fitted into a polynomial equation, in which crack length *a_i_* is a function of the cycle times *N_i_*. For each *n* point before and after any data point *i*, a total of (2*n* + 1) continuous data points were fitted using a quadratic polynomial (Equation (3)). The number of *n* was set to 3 in this paper.

(3) $$a_{i}=b_{0}+b_{1}\left(\frac{N_{i}-C_{1}}{C_{2}}\right)+b_{2}{\left(\frac{N_{i}-C_{1}}{C_{2}}\right)}^{2}$$

The crack growth rate at *N_i_* is derived from Equation (3):

(4) $${\left(\frac{da}{dN}\right)}_{a_{i}}=\frac{b_{1}}{C_{2}}+\frac{2b_{2}\left(N_{i}-C_{1}\right)}{C_{2}^{2}}$$

According to GB/T 15970.6-2007 “Corrosion of metals and alloys—Stress corrosion testing—part 6: Preparation and use of pre-cracked specimens for tests under constant load or constant displacement” [16], the expression of the stress intensity factor range Δ*K* at the crack tip of the improved WOL specimen is shown in Equation (5).

(5) $${\left(\Delta K\right)}_{a_{i}}=\frac{\Delta P\cdot C_{3}(a_{i}/W)}{B\cdot \sqrt{a_{i}}}$$

where *C*_3_ is dimensionless flexibility, a function of *a*/*W*, as shown in Equation (6).

(6) $$C_{3}\left(\frac{a}{W}\right)=\left[30.96\left(\frac{a}{W}\right)-195.8{\left(\frac{a}{W}\right)}^{2}+730.6{\left(\frac{a}{W}\right)}^{3}-1186.3{\left(\frac{a}{W}\right)}^{4}+754.6{\left(\frac{a}{W}\right)}^{5}\right]$$

After obtaining the ${\left(da/dN\right)}_{i}$ and ${\left(\Delta K\right)}_{i}$, the Paris expression of crack growth rate (Equation (7)) could be fitted, where *C* and *n* are constants determined by the material.

(7) $$da/dN=C{\left(\Delta K\right)}^{n}$$

### 2.4. Molecular Dynamics Simulation

**Figure 2:**
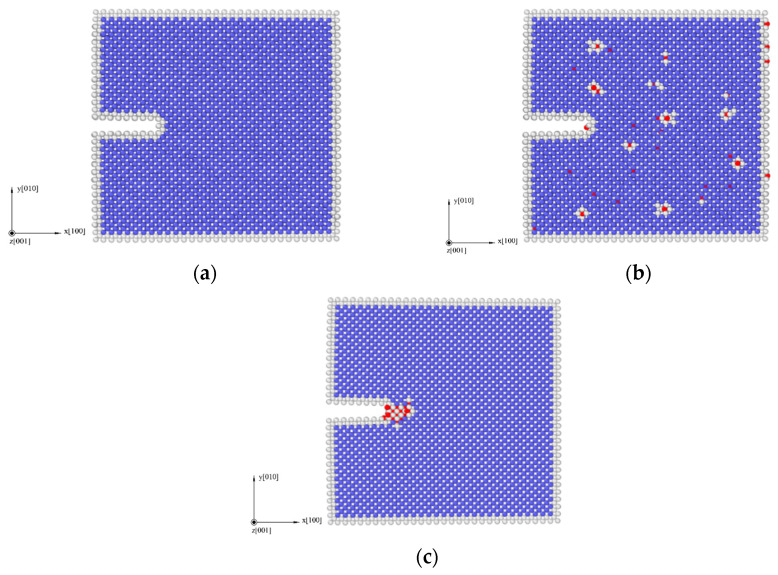
Molecular dynamics models (α-Fe in blue, hydrogen in red): (**a**) hydrogen-free model; (**b**) uniformly distributed hydrogen model; (**c**) hydrogen concentrated at the crack tip.

In order to better reveal the mechanism of corrosion fatigue crack propagation from a microscopic perspective, LAMMPS(8Feb2023-MPI, Sandia Natl Labs, Albuquerque, USA) [18], a molecular dynamics software, was used to simulate crack growth processed under different corrosion conditions. Three molecular dynamics models were established, namely: hydrogen-free condition (to simulate un-corroded material), uniformly distributed hydrogen condition (to simulate pre-corroded material, which has a uniform hydrogen distribution), and hydrogen concentrated at the crack tip (to simulate the coupling interaction of the material under corrosion environment and applied load, which has a stress-induced hydrogen redistribution). These three models of molecular dynamics corresponded to the experiments under three different corrosion environments. Taking the hydrogen-free α-Fe model as an example, the axes were chosen to be *x*-[1 0 0], *y*-[0 1 0], and *z*-[0 0 1]. The periodic boundary condition was applied in the z direction, while free surfaces were used for the *x* and *y* directions (SSP). The dimensions were 30*a* × 30*a* × 6*a*, where *a* is the lattice constant of iron (*a* = 0.28553 nm). The crack in the model was introduced by deleting the atomic layer on the left side of the model. The width of the initial crack was 0.5 nm and the length was 1/4 of the length in the *x*-direction of the model. The overall structure of the three models is shown in Figure 2.

**Figure 3:**
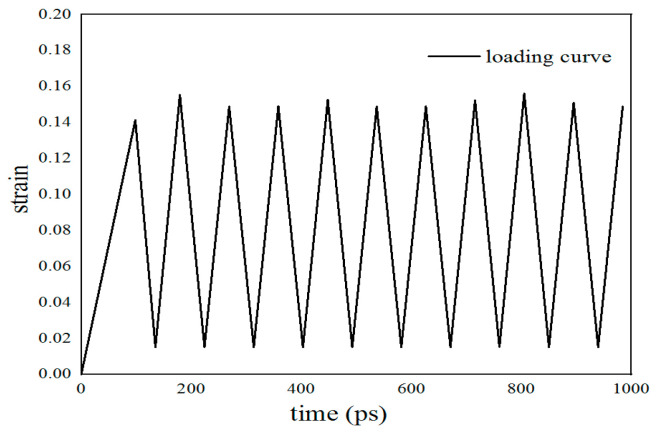
Load curve used in molecular dynamics simulation (T = 10.32 ps).

The model presented in this paper could be treated as a tiny unit of the whole α-Fe bulk material. The semi-empirical embedded-atom-method potential developed by Song [19] was used as the Fe-H interatomic interaction potential in the simulation. The embedded-atom-method (EAM) potential fitted by Ackland [20] was applied to the Fe-Fe interaction part of this potential function. The simulation process includes the following steps: The conjugate gradient method was used to minimize the energy of the model, and the model was relaxed by 100,000 steps under an isothermal–isobaric ensemble (NPT) to reach a new equilibrium state and a new configuration. The simulation temperature was kept at 300 K and the time step was set to 1.0 fs. The application of axial cyclic loads was modeled in a canonical ensemble (NVT). The simulation was performed by applying the fatigue load shown in Figure 3 (period of each cycle T = 10.32 ps, a total of ten cycles). Moreover, the integration algorithm used in the simulation was the default option verlet (velocity-Verlet). For the purpose of visualizing the defects and atomic configuration in the system, the software OVITO (3.9.1, Darmstadt, Germany) [21] was used. Defects were analyzed by mainly using common neighbor analysis (CNA) and the dislocation extraction algorithm (DXA).

## 3. Experimental and Simulation Results

### 3.1. da/dN-*Δ*K Curves under Different Experimental Conditions

**Figure 4:**
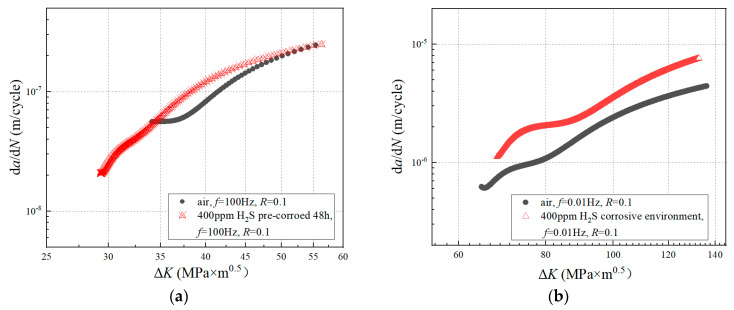
d*a*/d*N-*Δ*K* curves of different experimental conditions: (**a**) un-corroded and pre-corroded specimen under the loading condition of stress ratio *R* = 0.1, loading frequency *f* = 100 Hz; (**b**) un-corroded and corrosion fatigue specimen under the loading condition of stress ratio *R* = 0.1, loading frequency *f* = 0.01 Hz.

The d*a*/d*N* and Δ*K* under each stress cycle were calculated by using Equations (4) and (5), respectively. The d*a*/d*N*-Δ*K* curve is shown in log-log coordinates. The stable stage of the crack growth curve was selected to fit the parameters *C* and *n* in the Paris expression by using the least squares method. The d*a*/d*N*-ΔK curves under different test conditions are shown in Figure 4. The Paris expressions are shown in Table 3.

### 3.2. Simulation Results of Molecular Dynamics

**Figure 5:**
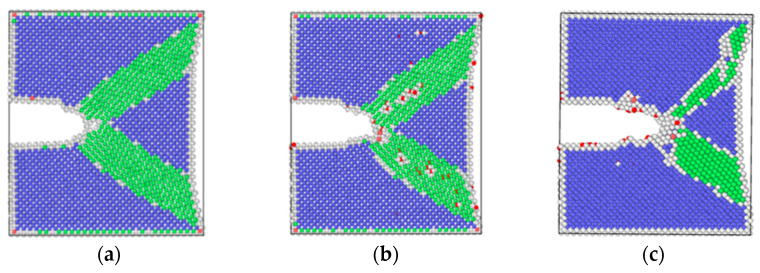
Crack propagation of three models: (**a**) hydrogen-free; (**b**) uniformly distributed hydrogen; (**c**) hydrogen concentrated at the crack tip.

As shown in Figure 5, by means of molecular dynamics simulation, the changes in microstructure of the hydrogen-free condition, uniformly distributed hydrogen condition, and hydrogen concentrated at the crack tip were obtained.

**Figure 6:**
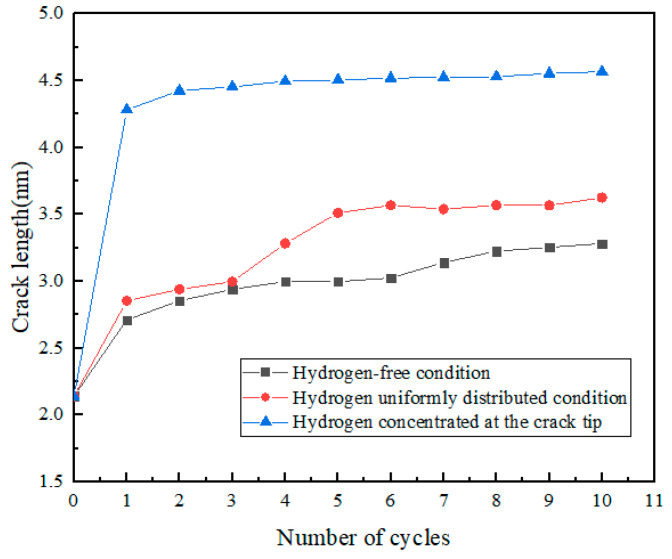
The relationship between cycle number and crack growth length under different environments.

The crack lengths for each cycle under different conditions were extracted and plotted, as shown in Figure 6.

## 4. Analysis and Discussion

### 4.1. Effect of Pre-Corrosion on Crack Growth Rate

**Figure 7:**
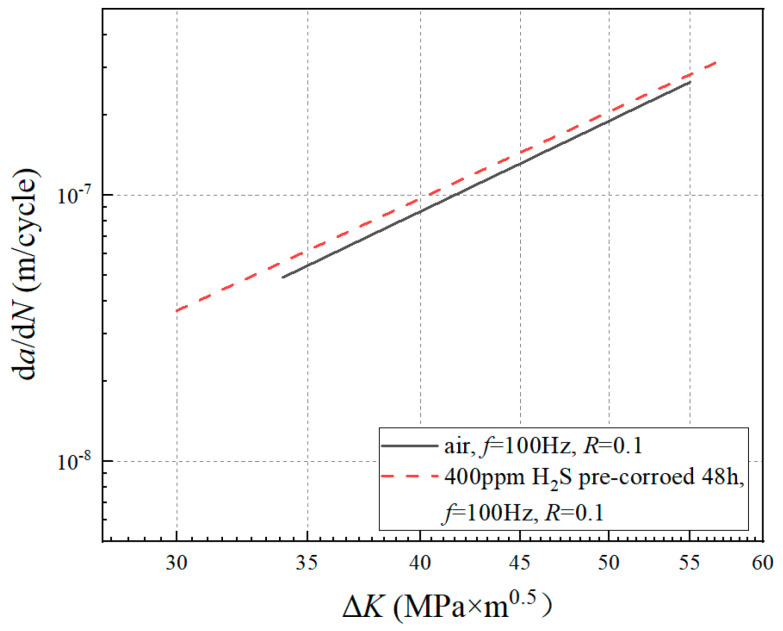
Crack growth curves before and after pre-corrosion (*R* = 0.1).

The comparison of crack growth rates of pre-corroded and non-corroded specimens is shown in Figure 7. It can be seen that the crack growth rate of the pre-corroded specimen is slightly higher than that of the non-corroded specimen at the initial stage. The two differ by about a factor of 1.26 when Δ*K* = 30. However, with the increase in Δ*K*, the gap between the two gradually decreases. The reason is that the hydrogen in the corrosion solution enters into the specimen through diffusion during the pre-corrosion process. The hydrogen diffusion behavior can be described by the following formula:

(8) $$\frac{\partial c}{\partial t}=D\nabla^{2}c-\frac{Dc}{RT}{\bar{V}}_{H}\nabla^{2}\sigma_{h}-\frac{Dc}{RT}-\nabla c\nabla \sigma_{h}$$

where *c* is the concentration of dissolved hydrogen in steel, *D* is the diffusion coefficient of hydrogen, *t* is the hydrogen diffusion time, ${\bar{V}}_{H}$ = 2.0 × 10^3^ mm^3^/mol is the partial molar volume, *R* = 8.314 J/(mol·K) is the gas molar constant, T is the Kelvin temperature, ∇c is the hydrogen concentration gradient, and $\nabla \sigma_{h}$ is the hydrostatic stress gradient.

Pre-corrosion could lead to the reduction in intergranular strength. Fatigue cracks were more easily formed at defects and corrosion pits, which accelerated the formation and propagation of fatigue cracks, resulting in the reduction in fatigue properties of the structures [22]. The results obtained in this paper show that pre-corrosion treatment increases the crack propagation rate and decreases the fatigue resistance of the material, which are similar to those described in the literature [22]. That is because hydrogen sulfide formed a sulfide film on the surface of the steel, which increases the self-corrosion potential of the specimen and promotes the cathodic reaction. On the surface of steel, H_2_S and its ionized S^2−^ and HS^−^ in water have an adsorption characteristic, which can inhibit the reaction of the atomic hydrogen of the cathode to combine into H_2_ so the atomic hydrogen can penetrate the material, which increases the brittle fracture sensitivity of steel.

However, there was no external load involved in the pre-corrosion process. The total amount and the permeation depth of atomic hydrogen were limited. Therefore, the crack growth rate of the pre-corrosion specimen was slightly increased. Meanwhile, due to the fatigue crack growth experiment that was tested in air after pre-corrosion, there was a certain phenomenon of hydrogen escape. Therefore, as the experiment progressed, the gap between the two curves gradually decreased.

**Figure 8:**
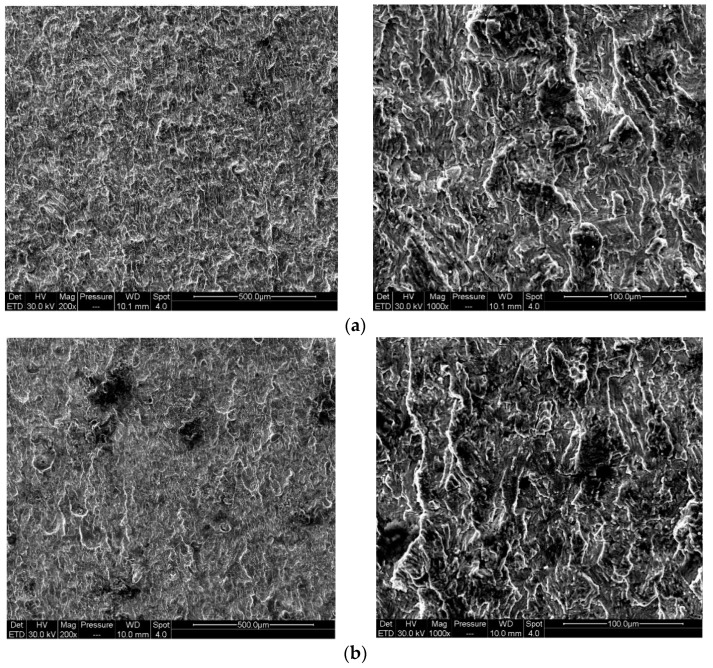
Fracture morphology under different corrosion environments: (**a**) fatigue crack growth fracture morphology in non-corrosive environment; (**b**) fatigue crack growth fracture morphology after pre-corrosion.

The morphologies of the main fracture surface under different corrosion conditions are shown in Figure 8. 

It can be seen from the figure that the fracture surfaces of the pre-corroded and the un-corroded specimen were basically the same, which indicated that the two conditions had similar fracture mechanisms. The obvious dimples on the fracture surfaces showed a strong ductile fracture characteristic, and the corrosion effect was small. This was because the concentration of hydrogen diffused into the specimen was limited. The corrosion degree of the pre-corroded crack tip was low, and the fracture properties of the material were not significantly deteriorated.

**Figure 9:**
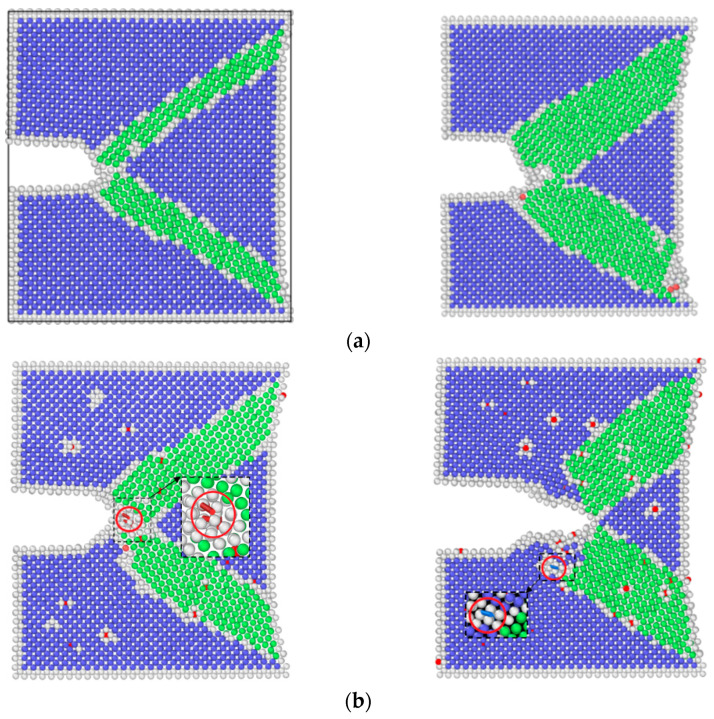
Dislocation emission during crack propagation: (**a**) hydrogen-free; (**b**) uniformly distributed hydrogen.

The results of molecular dynamics simulation showed that the crack growth of the uniformly distributed hydrogen model under the same cycle was slightly greater than that of the hydrogen-free model, while this result was in agreement with the experimental results. As can be seen from the uniformly distributed hydrogen model in Figure 5, hydrogen atoms were enriched near the crack tip, which was a high-stress region. This was due to the stress-induced hydrogen redistribution. The presence of hydrogen would promote the dislocation of the crack tip. The phase transformation and dislocation emission of the molecular dynamics model during crack propagation are shown in Figure 9. It can be seen that the plastic deformation mechanism of the hydrogen-free model was mainly a phase transition. For the uniformly distributed hydrogen model, the overall phase transformation of the model was similar to that of the hydrogen-free model. However, a small number of dislocations appeared at the crack tip during the crack propagation process. The plugging of the dislocations would lead to stress concentration, which would result in an increase in crack propagation.

### 4.2. Effect of Hydrogen Corrosion Environment on Crack Growth Rate

**Figure 10:**
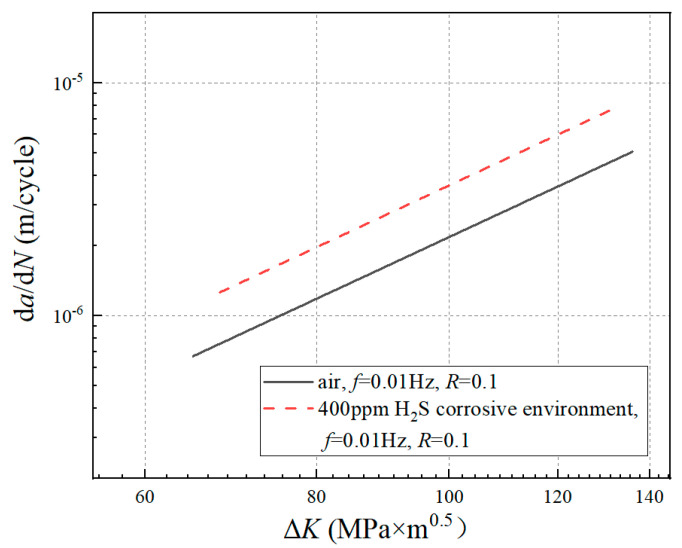
Crack growth rate curves under different corrosion modes (*R* = 0.1).

The difference in the FCGR between the corrosion solution and air under the same frequency, stress ratio, and maximum load is shown in Figure 10. It can be seen that the FCGR obtained from the corrosion solution was obviously higher than that in air. The two conditions differed by about a factor of 4. Meanwhile, the difference did not change with the increase in ΔK.

**Figure 11:**
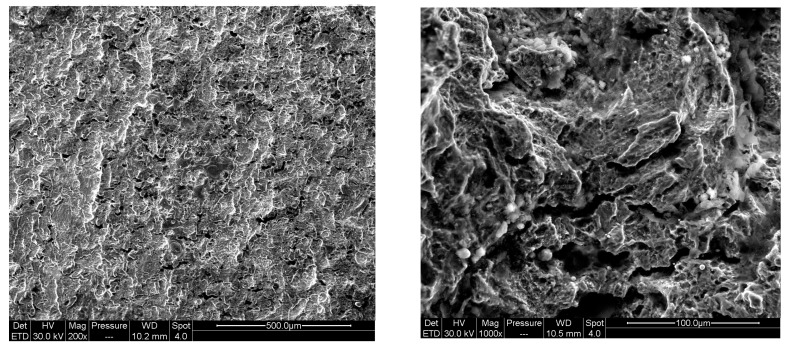
Fatigue crack growth fracture morphology in corrosion environment.

When the experiment was carried out in the corrosion solution, as the hydrogen atoms entered into the metal continuously, the total amount of hydrogen atoms in the specimen was much higher than that of the pre-corroded specimen. The plastic zone caused by the applied load was formed at the crack front, and hydrogen accumulated at the crack tip, enhanced the local plasticity, and caused the hydrogen embrittlement. The plastic deformation and high stress could easily promote the crack nucleation and propagation, which led to the deterioration of the fracture mechanical properties. Along with the crack growth, the size of plastic zone became larger, the degree of plastic deformation became greater, and the FCGR accelerated [23,24,25,26]. It can be seen from Figure 11 that, for the corrosion fatigue specimen, the dimples could still be observed on the main fracture surface while demonstrating the characteristics of cleavage fracture. Many micropores and certain corrosion products were found from the fracture, which indicated that the corrosion effect was strong in this condition. Meanwhile, the tendency of local corrosion and uniform corrosion was enhanced, showing that corrosion factors play an important role in the fracture process.

**Figure 12:**
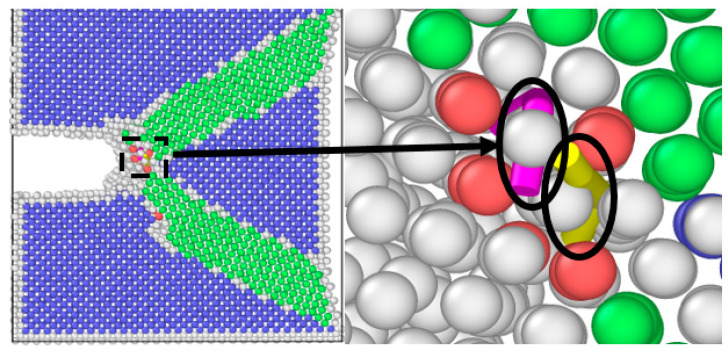
Dislocation emission from a hydrogen-containing α-Fe model at the crack tip.

As shown in Figure 12, the same law was also shown from the molecular dynamics simulation. The crack length of the hydrogen concentrated at the crack tip model under each cycle was greater than that of the hydrogen-free model. There were two diffusion modes of hydrogen atoms near the crack tip, the first was normal diffusion, that is, hydrogen atoms were spread between lattice gaps. According to the relevant literature [27], the diffusion path of hydrogen atoms in bcc iron was from the tetrahedral gap to neighboring tetrahedral gap, that is, T→T. The second was abnormal diffusion, in which hydrogen diffuses through material defects (such as grain boundaries, dislocations, vacancies). However, defects had various effects on the diffusion behavior of hydrogen. It might be a trap for hydrogen capture or a channel for hydrogen diffusion.

It could be seen from the atomic configuration that a large amount of hydrogen accumulated around the crack tip. Hydrogen promoted the emission, proliferation, and movement of the dislocation. Dislocations accumulated at the tip of the crack, so the plastic deformation mechanism during the crack propagation process was dominated by dislocations. The dislocation had a certain width, which can be considered a pipe, and hydrogen atoms diffused rapidly along with the dislocation pipe, aggravated hydrogen embrittlement, and caused brittle fracture of the material. This phenomenon led to a dramatic increase in the crack growth rate when the specimen was tested in the hydrogen corrosion environment.

In the process of corrosion fatigue, the crack tip was opened and closed repeatedly under cyclic loading. As the crack propagated, new crack surfaces were constantly created at the crack tip. The fresh metal was exposed to the corrosion solution, the potential was different from the corroded metal, and this phenomenon would form a micro-battery at the crack tip. So, the content of hydrogen ions at the crack tip increased greatly. The cyclic frequency of corrosion fatigue was 0.01 Hz; when the crack opened, the tip had enough time to make full contact with the corrosive solution, and a large amount of hydrogen entered the metal through absorption and diffusion. With the decrease in frequency, the degree of plastic deformation at the crack tip was sufficient. The probability of dislocation at the crack tip was increased. This allowed a high-speed passage for hydrogen to enter the interior of the material. Therefore, when the fatigue test was carried out in the corrosive environment, the hydrogen concentration of the specimen was much higher than that of the pre-corroded specimen, which led to a higher crack growth rate in the corrosive environment than in the pre-corrosive environment. In addition, sulfur ions as the toxic agent could prevent the overflow of hydrogen atoms, which further increased the concentration of hydrogen atoms. The low-frequency load combined with the hydrogen sulfide corrosion environment would amplify the effect of frequency on the crack growth rate. When the concentration of hydrogen in solution was fixed, the lower strain rate provided more time for hydrogen to penetrate into the crack tip. The penetration of hydrogen could lead to hydrogen embrittlement of the material. It accelerated the fatigue crack growth of material [28].

From the view of mechanical factors, the stress state of the crack tip was triaxial tension, which is the most vulnerable state. The high-hydrostatic-stress-induced hydrogen enrichment. Moreover, the holes, hydrogen traps, etc., caused by plastic deformation of the crack tip also gathered a large amount of hydrogen. This resulted in an increased degree of corrosion at the crack tip, which was reflected in a much higher rate of the fatigue crack growth in the corrosion environment.

### 4.3. Effect of Frequency on Crack Growth Rate

**Figure 13:**
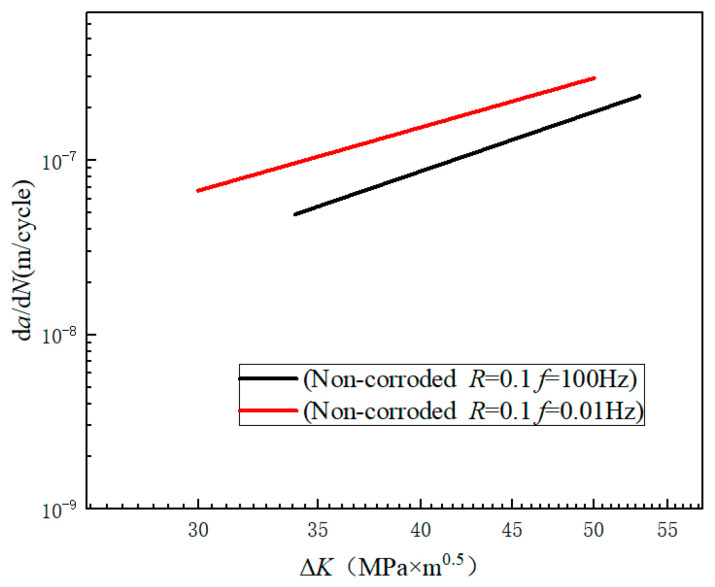
Crack growth rates at different frequencies.

It can be seen from Figure 13 that for the fatigue test in air, the fatigue crack growth curve obtained under the condition of low frequency (0.01 Hz) was higher than that of high frequency (100 Hz). Combined with Table 3, with the same *ΔK* (*ΔK* = 50), the crack growth rate was 1.5 times faster when the frequency changed 10,000 times. The main reason for this phenomenon was that with the decrease in loading frequency, the strain rate of the crack tip decreased. The deformation of the crack tip was more complete. The lower the frequency, the longer the fresh metal at the crack tip was exposed to the air, resulting in a thicker oxide film. The oxide film would hinder the reverse slip of the crystal, and result in an increased crack growth rate [29,30,31].

At the same time, it can be seen from Table 3 that when the loading frequency was fixed, the test environment had little effect on the crack growth rate parameter *n* in the Paris expression. When the frequency changed, even if the test environment was the same as air, the parameter *n* still changed significantly (from 3.513 to 2.911). This indicates that the frequency was the main factor affecting the slope of the fatigue crack growth curve. This is similar to the result obtained in the literature [32]: the lower the frequency, the smaller the value of parameter *n*. This shows the nonlinear effect of frequency on crack growth rate.

**Figure 14:**
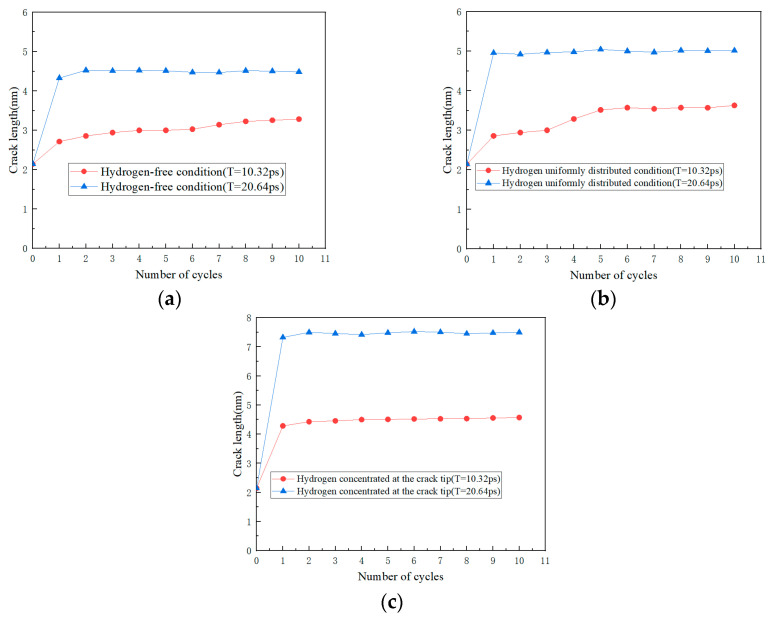
The relationship between cycle number and crack growth length at different frequencies (high-frequency T = 10.32 ps and low-frequency T = 20.64 ps): (**a**) hydrogen-free; (**b**) uniformly distributed hydrogen; (**c**) hydrogen concentrated at the crack tip.

In order to better study the effect of frequency on crack propagation, molecular dynamics simulations with different frequencies were performed. The crack lengths at three models in different frequencies (high-frequency T = 10.32 ps and low-frequency T = 20.64 ps) were extracted and plotted, as shown in Figure 14. The difference in frequency is achieved by modifying the loading rate to achieve different periods of the entire cycle. 

**Figure 15:**
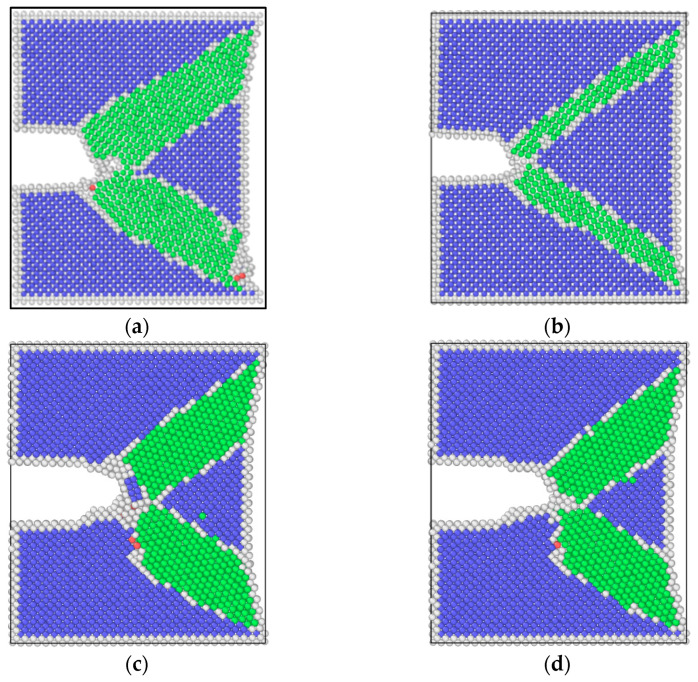
Atomic configuration diagrams of the hydrogen-free model: (**a**) the maximum load in a cycle, high-frequency (T = 10.32 ps); (**b**) the minimum load in a cycle, high-frequency (T = 10.32 ps); (**c**) the maximum load in a cycle, low-frequency (T = 20.64 ps); (**d**) the minimum load in a cycle, low-frequency (T = 20.64 ps).

Atomic configuration diagrams of the hydrogen-free model under different frequencies are shown in Figure 15. It can be seen that regardless of the frequency, the plastic deformation mechanism of the hydrogen-free model is dominated by phase transformation. Considering that the maximum loads of two loading conditions are the same, the sizes of the phase transition region obtained from the maximum load under high and low frequencies are similar. However, atomic configuration diagrams obtained from the minimum load show a clear difference; the phase transition region of low frequency is much larger than that of high frequency. The phenomenon is due to the low strain rate, which makes the plastic deformation more sufficient and accelerates the FCGR. 

**Figure 16:**
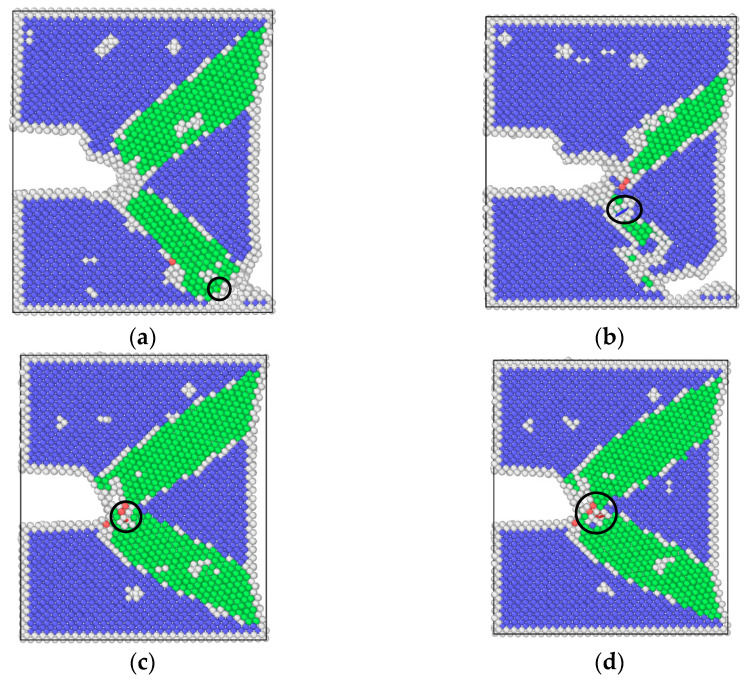
Dislocation distribution extracted from the uniformly distributed hydrogen model: (**a**) the maximum load in a cycle, high-frequency (T = 10.32 ps); (**b**) the minimum load in a cycle, high-frequency (T = 10.32 ps); (**c**) the maximum load in a cycle, low-frequency (T = 20.64 ps); (**d**) the minimum load in a cycle, low-frequency (T = 20.64 ps).

**Figure 17:**
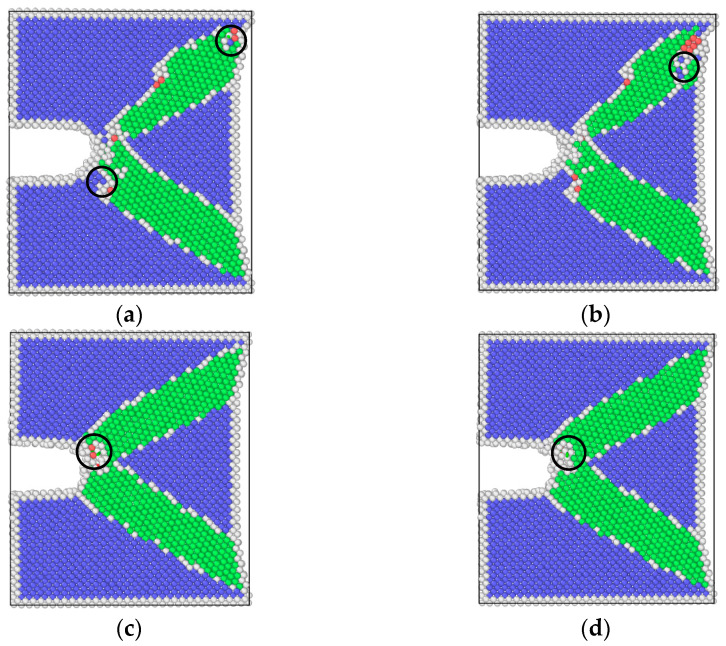
Dislocation distribution extracted from the hydrogen concentrated at the crack tip model: (**a**) the maximum load in a cycle, high-frequency (T = 10.32 ps); (**b**) the minimum load in a cycle, high-frequency (T = 10.32 ps); (**c**) the maximum load in a cycle, low-frequency (T = 20.64 ps); (**d**) the minimum load in a cycle, low-frequency (T = 20.64 ps).

Figure 16 and Figure 17 show the dislocation distributions extracted from the uniformly hydrogen distribution model and hydrogen concentrated at the crack tip model. It can be seen that at the maximum load point, dislocations begin to emit at the crack tip, but the propagation behaviors of dislocations are different at different frequencies. The atomic configuration diagrams obtained from the high-frequency loading condition show that the dislocation propagates away from the crack tip with the decrease in load, and the number of dislocations also decreases. The atomic configuration diagrams obtained from high frequency show dislocations obviously plugging at the crack tip. This phenomenon shows the coupling effect of the low strain rate and hydrogen. Dislocation plugging caused stress concentration, enhanced the passivation of the crack tip, and made the crack easier to propagate.

## 5. Conclusions

In this paper, the expression of the fatigue crack growth rate of 4130X material under different frequencies and corrosion states is obtained through experiments. The microscopic mechanism of crack growth under different corrosion states is obtained through molecular dynamics simulation.

The fatigue crack growth rate of 4130X material increases by 1.26 times after 48 h of pre-corrosion in a 400 ppm H_2_S environment. However, the difference decreases with the increase in ΔK. The morphology of both fractures shows that ductile fracture is the main fracture mechanism. Molecular dynamics simulation reveals that the plastic deformation mechanism of the crack tip in air is mainly transformation. Hydrogen introduced by pre-corrosion causes a small number of dislocations at the crack tip.

The corrosion fatigue crack growth rate of 400 ppm H_2_S is four times faster than that of air under the same loading condition. The fracture shows a strong corrosion effect. Molecular dynamics simulation shows that a large amount of hydrogen is accumulated at the crack tip. It promotes the emission, proliferation, and plugging of dislocation. During crack growth, the plastic deformation mechanism is dominated by dislocation. The coupling of low frequency and the corrosive environment intensifies the hydrogen embrittlement of the crack tip.

The crack growth rate of different frequencies (100 Hz, 0.01 Hz) in the air is compared. The frequency is reduced by 10,000 times, and the crack growth rate is only 1.5 times faster. Frequency is the main factor affecting parameter n in the Paris expression. The molecular dynamic simulation shows that low frequency promotes crack tip propagation.

## Figures and Tables

**Table 1:** Main chemical composition of 4130X.

Material	C	Si	Mn	Cr	Mo	P, S	Cu
4130X	0.26–0.34	0.17–0.37	0.40–0.70	0.80–1.10	0.15–0.25	≤0.035	≤0.30

**Table 2:** Mechanical properties of 4130X.

Material	*R*_m_/MPa	*R*_P0.2_/MPa	*E*/GPa	*A*/%	*Z*/%
4130X	777	624	209	14.23	52.38

**Table 3:** Expression of crack growth rate.

Loading Condition	Corrosion Condition	Crack Propagation Rate Expression
*f* = 100 Hz, *R* = 0.1	Un-corroded	d*a*/d*N* = 2.043 × 10^−13^(∆*K*)^3.513^
	Pre-corroded	d*a*/d*N* = 3.900 × 10^−13^(∆*K*)^3.368^
*f* = 0.01 Hz, *R* = 0.1	Un-corroded	d*a*/d*N* = 3.360 × 10^−12^(∆*K*)^2.911^
	Corrosion fatigue	d*a*/d*N* = 1.885 × 10^−11^(∆*K*)^2.640^

## Data Availability

The data presented in this study are available upon request from the corresponding author.
